# The AT1 Receptor Antagonist Losartan Does Not Affect Depressive-Like State and Memory Impairment Evoked by Chronic Stressors in Rats

**DOI:** 10.3389/fphar.2019.00705

**Published:** 2019-06-21

**Authors:** Willian Costa-Ferreira, Gessynger Morais-Silva, Lucas Gomes-de-Souza, Marcelo T. Marin, Carlos C. Crestani

**Affiliations:** ^1^School of Pharmaceutical Sciences, São Paulo State University (UNESP), Araraquara, Brazil; ^2^Joint UFSCar-UNESP Graduate Program in Physiological Sciences, São Carlos, Brazil

**Keywords:** losartan, depression, locomotion, anhedonia, memory, restraint stress, chronic variable stress, rats

## Abstract

The present study investigated the effect of the treatment with the angiotensin II type 1 receptor (AT_1_) antagonist losartan in the depressive-like state and memory impairment evoked by exposure to either homotypic (i.e., repeated exposure to the same type of stressor) or heterotypic (i.e., exposure to different aversive stimuli) chronic stressors in rats. For this, male Wistar rats were subjected to a 10 days regimen of repeated restraint stress (RRS, homotypic stressor) or chronic variable stress (CVS, heterotypic stressor) while being concurrently treated daily with losartan (30 mg/kg/day, p.o.). Depressive-like state was evaluated by analysis of the alterations considered as markers of depression (decreased sucrose preference and body weight and coat state deterioration), whereas cognitive non-emotional performance was tested using the novel object recognition (NOR) test. Locomotor activity was also evaluated in the open field test. Both RRS and CVS impaired sucrose preference and caused coat state deterioration, whereas only CVS impaired body weight gain. Besides, RRS impaired short-term memory (but not long-term memory) in the NOR test. Neither depressive-like state nor memory impairment evoked by the chronic stressors was affected by the treatment with losartan. Nevertheless, CVS increased the locomotion, which was inhibited by losartan. Taken together, these results provide evidence that the chronic treatment with losartan does not affect the depressive-like state and memory impairment evoked by either homotypic or heterotypic chronic stress regimens in rats.

## Introduction

Clinical and preclinical studies have provided evidence of the emotional stress as a prominent factor predisposing to depression (Mazure, 1998; Hammen, 2005; Willner, 2005; Grippo and Johnson, 2009; Buynitsky and Mostofsky, 2009; Liu and Alloy, 2010; Carnevali et al., 2017). Exposure to adverse events is also related to learning and memory impairment (Buynitsky and Mostofsky, 2009; Wolf, 2009; Quaedflieg and Schwabe, 2018). In this sense, several studies have indicated that the impact of stress is determined by characteristics of the stressor stimulus, such as chronicity, predictability, controllability, and severity (Koolhaas et al., 2011; Crestani, 2016). Studies in rodents have explored the influence of predictability by comparing the effect of chronic stressors involving daily exposure to the same stressor (i.e., homotypic/predictable) versus different aversive stimuli (i.e., heterotypic/unpredictable) (Magariños and McEwen, 1995; Marin et al., 2007; Kopp et al., 2013; Pastor-Ciurana et al., 2014; Duarte et al., 2015; Costa-Ferreira et al., 2016). These studies have typically used protocols of repeated restraint stress (RRS) as homotypic stressor, whereas the chronic variable stress (CVS) is often employed as a heterotypic stressor (Crestani, 2016). Studies comparing CVS and RRS have demonstrated that the former evokes more severe changes on somatic parameters (e.g., adrenal hypertrophy and thymic involution) and hypothalamic–pituitary–adrenal (HPA) axis activity (Magariños and McEwen, 1995; Haile et al., 2001; Marin et al., 2007; Kopp et al., 2013; Pastor-Ciurana et al., 2014; Costa-Ferreira et al., 2016), which is possibly related to the habituation process identified in the RRS as consequence of the repeated exposure to the same stressor (Herman, 2013; Crestani, 2016; McCarty, 2016). Differences in depression- and anxiety-like behaviors and memory are less understood since a limited number of studies compared the effects of RRS versus CVS on these behaviors. For the best of our knowledge, the only study comparing RRS and CVS on behavioral responses was a recent study from our group in which we identified increase in anxiety-like behaviors in female (but not male) rats exposed to either RRS or CVS (Vieira et al., 2018). However, differences in depression-like behaviors and memory have never been evaluated.

Angiotensin II (Ang II) is an active peptide of the renin-angiotensin system (RAS) that has been historically implicated in the cardiovascular and hydroelectrolytic control (Hall, 2003; Karnik et al., 2015). Several reports, however, have demonstrated that in addition to its formation and action in the circulation as a blood-borne hormone, Ang II is also synthetized within the central nervous system (Saavedra, 2005). Indeed, RAS components and Ang II receptors (i.e., AT_1_ and AT_2_ receptors) were identified in limbic structures controlling stress responses (Wright and Harding, 2011; Bali and Jaggi, 2013). In this sense, previous studies have documented an involvement of the Ang II acting *via* activation of the AT_1_ receptor in the etiology of stress-evoked diseases (Watanabe et al., 1998; Saavedra et al., 2004; Saavedra et al., 2011; Bali and Jaggi, 2013; Ayyub et al., 2016; Fontes et al., 2016). The mechanisms related to involvement of Ang II/AT_1_ receptor in complications evoked by stress are not completely understood, but neuroinflammation, dysregulated hormonal and sympathetic responses, and oxidative stress might be involved (Saavedra et al., 2011; Labandeira-Garcia et al., 2017; Saavedra, 2017). Accordingly, the therapeutic use of AT_1_ receptor antagonists in the treatment of stress-related pathologies has been discussed (Quijano and Arango, 1979; Saavedra, 2005; Saavedra, 2017). However, the effects of AT_1_ receptor antagonists in behavioral responses to stress are still not fully understood.

Previous studies identified that systemic administration of AT_1_ receptor antagonists inhibited the anxiogenic-like effect evoked by both RRS and CVS (Pechlivanova et al., 2011; Ping et al., 2014; Ayyub et al., 2016; Wincewicz et al., 2016; Ranjbar et al., 2018). AT_1_ receptor antagonists also prevented the increase of immobility in the forced swimming test and tail-suspension test, as well as the decreased sucrose preference evoked by protocols of CVS in mice (Ping et al., 2014; Ayyub et al., 2016), thus indicating an antidepressant-like effect. However, depressive-like behaviors were only investigated in mice; and the effect of AT_1_ receptor antagonists in depressive-like effect to homotypic stressors has never been evaluated. Regarding stress-evoked memory impairment, previous studies identified that memory impairment in the novel object recognition (NOR) test and passive avoidance situation evoked by a RRS protocol were inhibited by treatment with AT_1_ receptor antagonists in rats (Braszko et al., 2013; Wincewicz and Braszko, 2014; Wincewicz et al., 2016). Treatment with AT_1_ receptor antagonist also inhibited impairment of spatial memory evoked by RRS in rats (Wincewicz and Braszko, 2015). Nevertheless, the influence of AT_1_ receptor antagonists in memory impairment evoked by heterotypic stressors has never been evaluated.

The results described above indicate important effects of AT_1_ receptor antagonists in behavioral changes evoked by chronic stress. However, some important issues regarding the effects of AT_1_ receptor antagonists in stress-evoked depressive-like state and memory impairment are still to be addressed, including: i) comparison of the behavioral responses evoked by homotypic versus heterotypic stressors; ii) evaluation of depressive-like responses to stress in rats, including analysis of behaviors other than anhedonia and despair (e.g., self-care); iii) investigation of depressive-like effect evoked by homotypic stressors; and iv) evaluation of memory impairment evoked by heterotypic stressors. Therefore, in the present study we attempted to investigate the effect of the systemic treatment with the AT_1_ receptor antagonist losartan in depressive-like state and memory impairment evoked by exposure to either the heterotypic stressor CVS or the homotypic stressor RRS. The potential influence of unspecific effects of the stressors and/or losartan treatment on locomotor activity was also evaluated.

## Materials and Methods

### Animals

One hundred twenty 60-day-old male Wistar rats weighing 200 ± 10 g were used in this study. The animals were obtained from the animal breeding facility of the São Paulo State University-UNESP (Botucatu, SP, Brazil), and were housed in collective plastic cages (four rats/cage). The animals remained in temperature-controlled room at 24°C with light–dark cycle 12:12 h (lights on between 7:00 a.m. and 7:00 p.m.) with free access to water and standard laboratory food in the Animal Facility of the Laboratory of Pharmacology-UNESP (Araraquara, SP, Brazil). The procedures and protocols were approved by the local Ethical Committee for Use of Animals (approval # 32/2014), which complies with Brazilian and international guidelines for animal use and welfare.

### Chronic Stress Protocols

The protocols of RRS and CVS were based on previous studies from our group, which we reported behavioral, neuroendocrine, cardiovascular, and somatic changes following exposure to these chronic stressors (Marin et al., 2007; Cruz et al., 2012; Duarte et al., 2015; Costa-Ferreira et al., 2016; Vieira et al., 2018). In this sense, RRS was used as a homotypic stressor and CVS was chosen as a heterotypic stress regimen. For the RRS, animals were restrained in opaque plastic cylinders (15 cm length and 5.5 cm internal diameter) 1 h daily (starting always at 9:00 a.m.) for 10 consecutive days. For the CVS, animals were exposed to different stressors in a variable schedule for 10 consecutive days, according to protocol employed in our laboratory (**Table 1**) (Duarte et al., 2015; Costa-Ferreira et al., 2016; Vieira et al., 2018). All stress sessions were performed in an adjacent room to the animal facility. RRS and CVS started simultaneously, and during this period, animals of the control groups were left undisturbed, except for cleaning the cages and pharmacological treatment, in the animal facility.

**Table 1:** Protocol of CVS.

Day	Stress type and schedule
1	10:00 AM, restraint stress, 60 min; 7:00 PM, humid sawdust, overnight
2	3:00 PM, cold (4°C) isolation, 60 min; 7:00 PM, lights on, overnight
3	12:00 AM, lights off, 180 min; 3:00 PM, swim stress, 4 min
4	7:30 AM, humid sawdust, all day; 7:00 PM, food/water deprivation, overnight
5	1:00 PM, swim stress, 3 min; 7:00 PM, isolation housing, overnight
6	2:00 PM cold (4°C) isolation, 15 min; 3:00 PM, lights off, 120 min
7	7:00 PM, humid sawdust and lights on, overnight
8	7:00 PM, isolation and food/water deprivation, overnight
9	4:00 PM, restraint stress, 60 min; 7:00 PM, lights on, overnight
10	8:00 AM, swim stress, 4 min; 10:00 h, restraint stress, 60 min

### Pharmacological Treatment

The selective AT_1_ receptor antagonist losartan was purchased from Sigma–Aldrich (St. Louis, MO, USA), and was diluted in saline solution (NaCl 0.9%). The pharmacological treatment with losartan (30 mg/kg/day) or vehicle (saline) started on the first day of the stress protocols and was continued daily for 10 consecutive days. The treatment was based on previous reports that losartan at this dose was effective in inhibiting the increase in plasma glucose, norepinephrine, epinephrine, and corticosterone levels; as well as the cardiovascular dysfunctions, evoked by stress in rats (Üresin et al., 2004; Costa-Ferreira et al., 2016). Losartan or vehicle was given once daily by gavage at 8:00 a.m. (Costa-Ferreira et al., 2016).

### Sucrose Consumption Test

The sucrose preference test was used as a behavioral test for evaluation of anhedonia. The protocol did not include periods of food and water deprivation (D’Aquila et al., 1997; Papp, 2012; Antoniuk et al., 2019). The animals were housed individually, and the test consisted of two phases: i) habituation and ii) testing. The habituation phase was performed in 2 days. On the first day, the animals were exposed to two drinking bottles containing sucrose (2%, v/v), which was placed at the beginning of the dark phase of the light/dark cycle (i.e., 7:00 p.m.) and kept for a period of 24 h (habituation: day 1). Thereafter, the two bottles containing sucrose were replaced by other two containing water, and the animals had access to bottles containing water for 24 h (habituation: day 2).

After completion of the habituation phase, the animals were tested for sucrose preference (testing phase). For this, two drinking bottles were offered at the beginning of the dark phase of the light/dark cycle (i.e., 7:00 p.m.): one containing sucrose solution (2%, v/v) and one containing water. Sucrose preference was calculated by weighting the bottles (values obtained in grams) at the beginning of the exposure and after 3 h (3 h sucrose preference) and 24 h (24 h sucrose preference). The percentage of sucrose preference was calculated as the ratio of sucrose solution consumed over the total amount of fluid consumed (water + sucrose solution) × 100.

To control liquid lost by spillage or evaporation, the weight of drinking bottles placed in empty cages at the same time as the solutions were offered to animals was evaluated, and the values were discounted from the amount consumed by the animals.

### Coat State Evaluation

Coat state deterioration has been described as a reliable and well-validated index of depressed-like state, which parallel symptoms identified in human depression of loss of motivation to maintain personal hygiene and self-care (Santarelli, 2003; Alonso et al., 2004; Nollet et al., 2013). Coat state was evaluated using a scale from 3 to 0, wherein 3 represents a healthy and well-cared fur while 0 represents a sick and dirty fur, with hair loss and piloerection. Intermediate states were scored as variations of 0.5 point. Coat state was evaluated in a blinded manner in the last day of the chronic stress protocols.

### Open Field Test

The open field (OF) test was used for evaluation of locomotion (Prut and Belzung, 2003; Grippo et al., 2014). The OF consisted of a PLEXIGLAS chamber measuring 54 cm (width) × 54 cm (length) × 30 cm (height). A central area in the middle of the arena measuring 24 cm (width) × 24 cm (length) was defined as an exposed field and is referred as “center.” Rats were individually placed in the middle of the arena and were allowed to explore freely the OF for 5 min. Analysis included measures of the distance travelled in central (central locomotion) and peripheral area (peripheral locomotion), as well as the total distance travelled (i.e., center + periphery) (total locomotion). All sessions were videotaped (Webcam LifeCam Cinema HD 720p Microsoft^®^ using software Microsoft LifeCam version 3.22) and analysis was realized in a blinded manner using the software ANY-maze^®^ (Stoelting, Wood Dale, Illinois, USA).

### Novel Object Recognition Test

The learning performance and non-emotional memory of the animals were examined through the NOR test, which was adapted from Carey et al. (2009). The NOR test is based on the spontaneous exploration of environment, with premise that animals spend more time exploring a new object than a familiar one (Antunes and Biala, 2012).

Initially, the animals were adapted for 5 min to the apparatus (the same apparatus described in the OF test) wherein the NOR test was performed. Twenty-four hours later, phase I started. At this stage, objects A and A’ (a pair of transparent rectangular glass bottles) were centered at the ends of the apparatus, 10 cm from the walls. In phase II, carried out 10 min later, the object A’ was replaced by a colorful cube of 7 cm height × 7 cm width, called object B, and the short-term memory (SM) was assessed. In phase III, performed 24 h after phase II, the long-term memory (LM) was evaluated on the same apparatus by changing the object B by a new object, a blue rectangle of 8 cm height × 10 cm width called object C. The same animals were evaluated for short- and long-term memory.

For each phase, the time the animals explored each object was recorded (Webcam LifeCam Cinema HD 720p Microsoft^®^ using software Microsoft LifeCam version 3.22), and the exploration was blindly analyzed using the software X-PloRat (version 2005, 1.1.0). For each object, the interaction period was defined as the time while the animal remained in physical contact with the object. Data were presented as the recognition index, which was determined by time spent on the new object divided by the time spent on both objects.

### Experimental Design

In each experiment, the rats were divided into six groups: i) control vehicle, ii) control losartan, iii) RRS vehicle, iv) RRS losartan, v) CVS vehicle, and vi) CVS losartan. The protocols of chronic stress and the pharmacological treatment with losartan started on the same day and continued for 10 consecutive days.

#### Experiment 1: Effects of Chronic Stress and/or Losartan Treatment in the Depression-Like Behaviors and Locomotion

The six experimental groups (n = 10/group) were subjected to the 10 days regimen of RRS or CVS while being concurrently treated daily with the selective AT_1_ receptor antagonist losartan (30 mg/kg/day, p.o.). Four evaluations were performed in the animals in this protocol. Coast state and body weight were evaluated on the 10^th^ day, after the last session of stress/treatment. On the 11^th^ day, the animals of all experimental groups were subjected to OF for evaluation of the locomotion. Lastly, the sucrose consumption test started at the night of the 11^th^ day. At the end of the experiments, the rats were euthanized *via* anesthetic overdose (urethane, 250 mg/ml/200 g body weight, i.p.).

#### Experiment 2: Effects of Chronic Stress and/or Losartan Treatment in the Short-Term and Long-Term Memory

Such as in the previous protocol, the six experimental groups (n = 9/group) were subjected to the 10 days regimen of RRS or CVS while being concurrently treated daily with losartan (30 mg/kg/day, p.o.), and the memory was evaluated in the NOR test. On the 11^th^ day, 24 h after the last session of stress/treatment, the animals of all experimental groups were allowed to habituate for 5 min to the apparatus wherein the NOR test was performed (the same apparatus used in the OF test). Twenty-four hours later, the animals were subjected to NOR test for evaluation of short-term memory. Long-term memory was assessed 24 h later. At the end of the experiments, the rats were euthanized *via* anesthetic overdose (urethane, 250 mg/ml/200 g body weight, i.p.).

### Data Analysis

Data were expressed as mean ± SEM. All data were analyzed using the software GraphPad Prism version 7.0 (GraphPad Software Inc., La Jolla, CA, USA). The data were analyzed using two-way ANOVA, with stress and pharmacological treatment as independent factors. When statistical differences were identified by two-way ANOVA, Bonferroni *post hoc* test was performed to assess specific differences between the experimental groups. P < 0.05 was assumed as significant.

## Results

### Effects of Chronic Stress and/or Losartan Treatment in the Depression-like Behaviors and Locomotion

**Figure 1:**
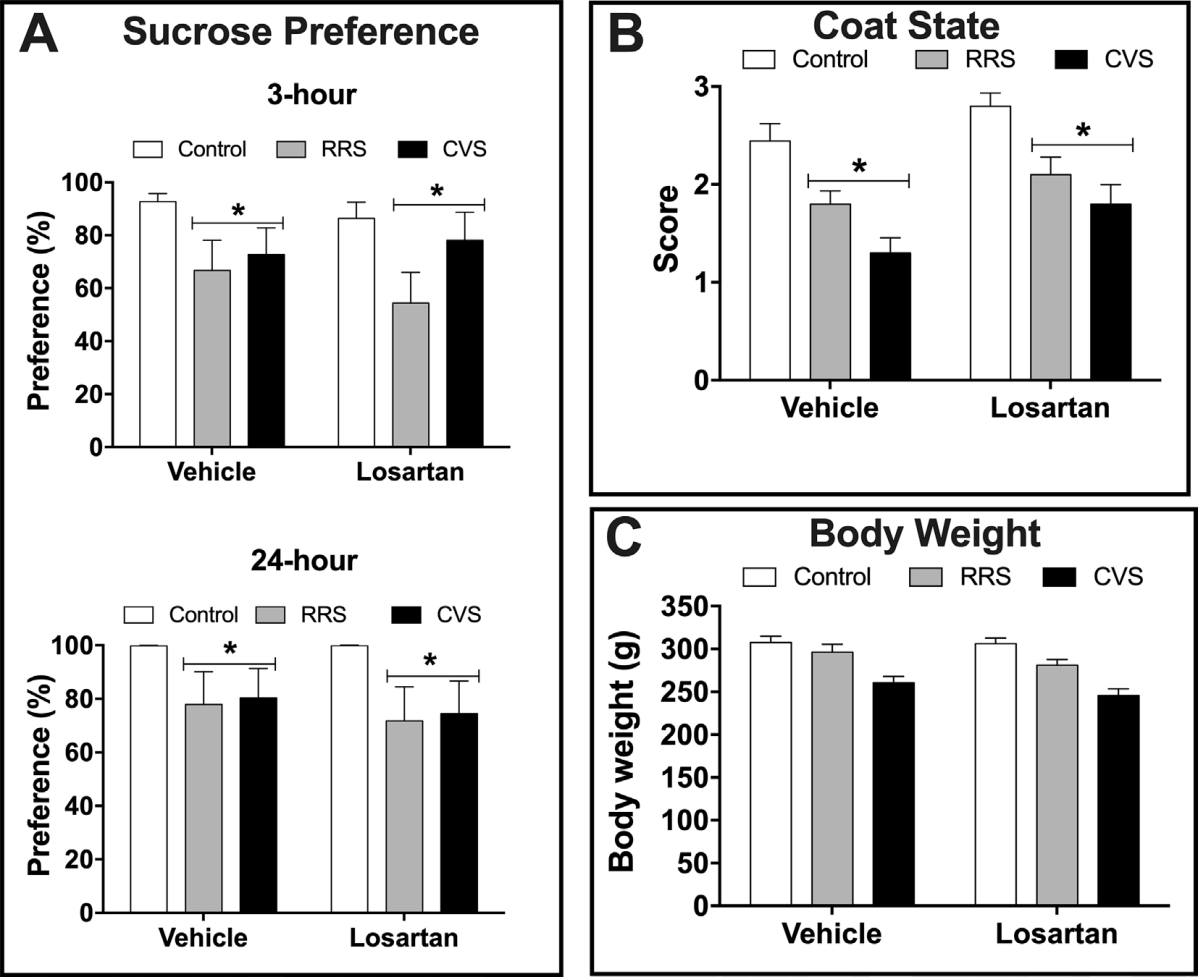
Depressive-like state in animals treated with either vehicle or losartan control (white bars) and subjected to RRS (gray bars) or CVS (black bars). **(A)** Sucrose preference (%) evaluated during 3 and 24 h. *P < 0.05 versus respective control group, two-way ANOVA (n = 10/group). **(B)** Coat state score. *P < 0.05 versus respective control group, two-way ANOVA (n = 10/group). **(C)** Body weight at the 10^th^ day of stress protocol. Two-way ANOVA (n = 10/group). The bars in all graphs represent the mean ± SEM.

*Depressive-like state*—Analysis of the sucrose preference indicated effect of stress (3 h: F_(2,54)_ = 4.93, P < 0.01; 24 h: F_(2,54)_ = 3.87, P < 0.02) in both 3 and 24 h consumption analysis, but without influence of losartan treatment (3 h: F_(1,54)_ = 0.33, P > 0.05; 24 h: F_(1,54)_ = 0.24, P > 0.05) and stress × treatment interaction (3 h: F_(2,54)_ = 0.46, P > 0.05; 24 h F_(2,54)_ = 0.06, P > 0.05) (**Figure 1A**). Analysis of the sucrose consumption (data not shown) also indicated effect of stress (3 h: F_(2,54)_ = 5.06, P < 0.009; 24 h: F_(2,54)_ = 4.29, P < 0.01) in analysis of 3 and 24 h, but without influence of losartan treatment (3 h: F_(1,54)_ = 0.07, P > 0.05; 24 h: F_(1,54)_ = 0.40, P > 0.05) and stress × treatment interaction (3 h: F_(2,54)_ = 0.82, P > 0.05; 24 h F_(2,54)_ = 0.65, P > 0.05).

Analysis of coat state deterioration indicated effect of stress (F_(2,54)_ = 21.51, P < 0.0001) and losartan treatment (F_(1,54)_ = 8.25, P < 0.005), but without stress × treatment interaction (F_(2,54)_ = 0.20, P > 0.05) (**Figure 1B**). Analysis of the body weight at the last day of stress protocols (i.e., 10^th^ day) indicated effect of stress (F_(2,54)_ = 25.61, P < 0.0001), but without effect of losartan treatment (F_(1,54)_ = 2.25, P > 0.05) and stress × treatment interaction (F_(2,54)_ = 0.42, P > 0.05) (**Figure 1C**).

**Figure 2:**
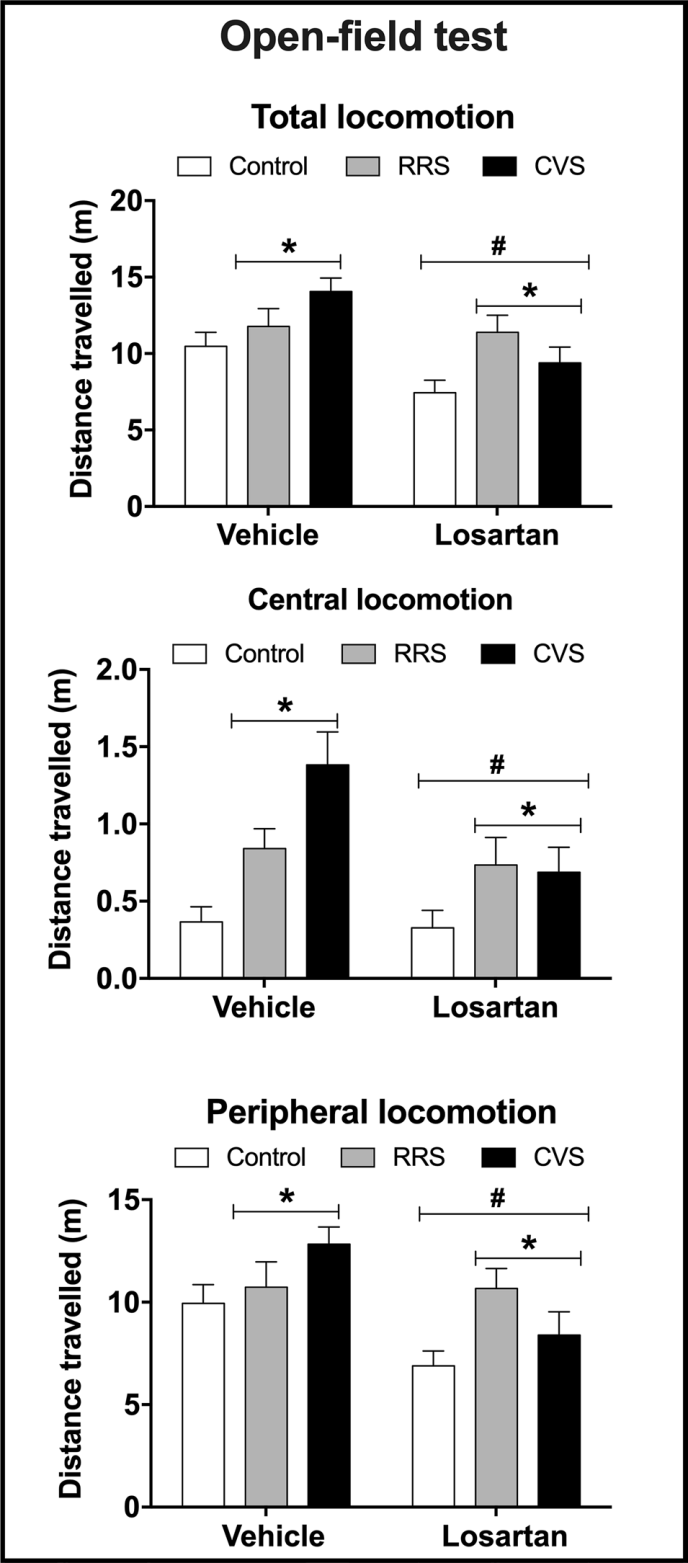
Total locomotion (distance travelled in the periphery + center, top graph) and distance travelled in the center (central locomotion, middle graph) and periphery (peripheral locomotion, bottom graph) in the open field apparatus in animals treated with either vehicle or losartan control (white bars) and subjected to RRS (gray bars) or CVS (black bars). The bars represent the mean ± SEM. *P < 0.05 vs respective control group, ^#^P < 0.05 vs respective vehicle groups. Two-way ANOVA followed by Bonferroni *post-hoc* test (n = 8–10/group).

*Locomotion*—Analysis of the total, central, and peripheral locomotion in the OF test indicated effect of stress (total: F_(2,54)_ = 4.77, P < 0.01; central: F_(2,54)_ = 9.49, P < 0.0004; peripheral: F_(2,54)_ = 3.28, P < 0.04) and losartan treatment (total: F_(1,54)_ = 10.63, P < 0.002; central: F_(1,54)_ = 4.75, P < 0.03; peripheral: F_(1,54)_ = 9.56, P > 0.003), but without stress × treatment interaction (total: F_(2,54)_ = 2.32, P > 0.05; central: F_(2,54)_ = 2.57, P > 0.05; peripheral: F_(2,54)_ = 2.55, P > 0.05) (**Figure 2**).

### Effects of Chronic Stress and/or Losartan Treatment in the Short-Term and Long-Term Memory

**Figure 3:**
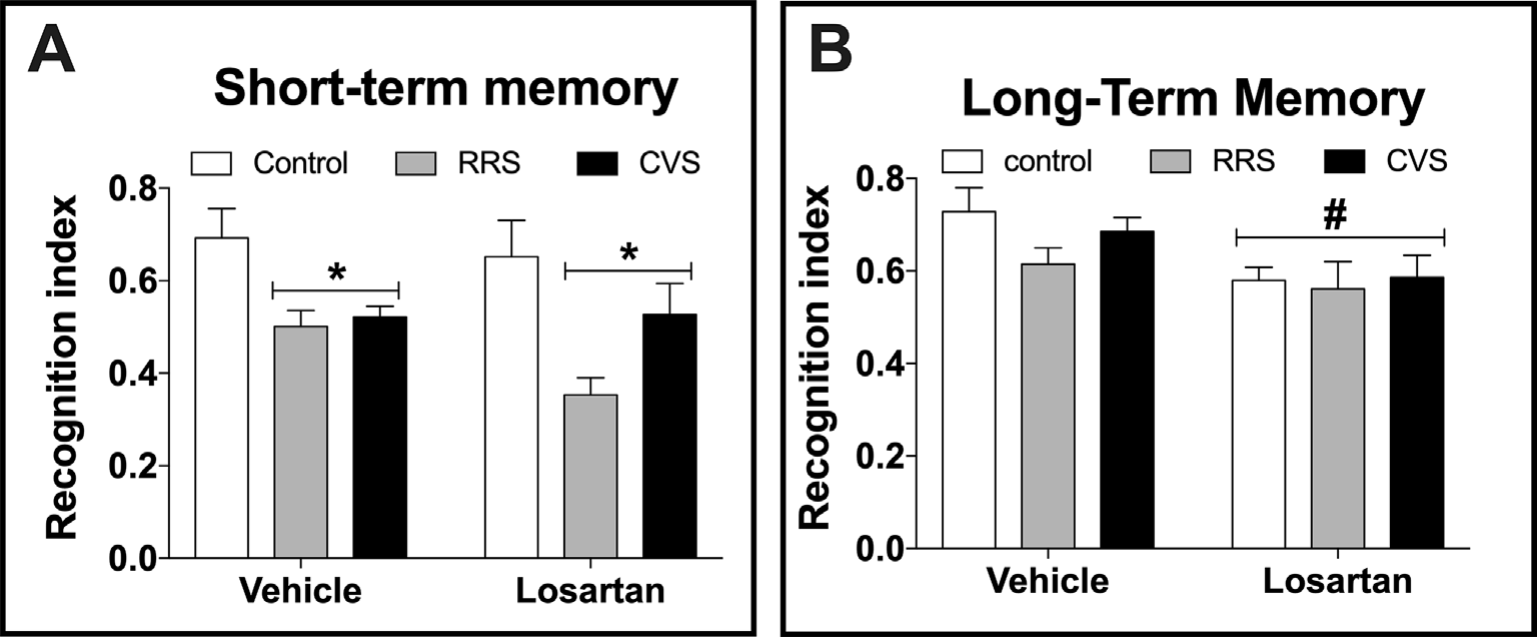
Cognitive non-emotional performance in the novel object recognition (NOR) test in animals treated with either vehicle or losartan control (white bars) and subjected to RRS (gray bars) or CVS (black bars). **(A)** Object discrimination rate at day 1 of NOR test (short-term memory). *P < 0.05 versus respective control group, two-way ANOVA (n = 9/group). **(B)** Object discrimination rate at day 2 of NOR test (long-term memory). ^#^P < 0.05 versus vehicle groups, two-way ANOVA (n = 9/group). The bars in all graphs represent the mean ± SEM.

Analysis of the short-term memory indicated effect of stress (F_(2,48)_ = 10.54, P < 0.0002), but without influence of losartan treatment (F_(1,48)_ = 0.16, P > 0.05) and stress × treatment interaction (F_(2,48)_ = 0.33, P > 0.05) (**Figure 3A**). Analysis of the long-term memory indicated effect of losartan treatment (F_(1,48)_ = 7.99, P < 0.006), but without influence of stress (F_(2,48)_ = 1.2, P > 0.05) and stress × treatment interaction (F_(2,48)_ = 0.58, p > 0.05) (**Figure 3B**).

## Discussion

The present study is the first to compare the effects of homotypic versus heterotypic chronic stressors in depression and memory. Our results are in line with previous evidence that both CVS and RRS evoked depressive-like state and memory impairment (Willner, 2005; Buynitsky and Mostofsky, 2009; Willner, 2017). Nevertheless, as stated above, previous studies comparing RRS vs CVS have demonstrated that increase in HPA axis activity, adrenal hypertrophy, and thymic involution are mainly observed after CVS exposure, whereas RRS minimally affects these parameters (Magariños and McEwen, 1995; Haile et al., 2001; Marin et al., 2007; Kopp et al., 2013; Pastor-Ciurana et al., 2014; Costa-Ferreira et al., 2016). These differences have been proposed to be related to the habituation process of the HPA axis activation identified in RRS, which is an important adaptive response that limits the long-term impact of chronic stressors (Herman, 2013; Crestani, 2016; McCarty, 2016). However, this habituation is more consistently observed in parameters related to HPA axis than other biological responses (Crestani, 2016). For instance, several studies have indicated that habituation of cardiovascular responses upon repeated exposure to restraint stress is limited or absent (McDougall et al., 2000; Conti et al., 2001; Daubert et al., 2012; Benini et al., 2019). Accordingly, similar cardiovascular and autonomic changes were identified following exposure to either RRS or CVS (Duarte et al., 2015; Costa-Ferreira et al., 2016; Vieira et al., 2018). Our results are further supported by evidence that RRS and CVS evoke similar morphological changes in limbic structures (Magariños and McEwen, 1995). Therefore, data reported here are consistent with the idea that habituation, which limits the long-term impact of stress (Herman, 2013; McCarty, 2016), is a specific response of some biological system rather than a general body response; so that some dysfunctions (e.g., depression and memory impairment) might be similarly evoked by both homotypic and heterotypic chronic stressors.

Evaluation of the depression-like state in the present study included analysis of a series of changes that are commonly used as markers of depression in rodents, such as decreased sucrose preference and body weight gain and coat state deterioration (Willner, 2005; Nollet et al., 2013). Treatment with losartan did not affect any of the depression-like responses evaluated in the present study. Our findings contrast with previous evidence that systemic treatment (p.o.) with valsartan inhibited the decrease of sucrose preference evoked by a CVS protocol in mice (Ping et al., 2014). Systemic treatment with either valsartan or irbesartan also inhibited the CVS-evoked increase of immobility in the forced swimming test and tail-suspension test in mice (Ping et al., 2014; Ayyub et al., 2016). To the best of our knowledge, present study is the first to investigate the effect of the treatment with an AT_1_ receptor antagonist in stress-evoked depression-like state in rats, so that discrepancy with previous studies might be explained by the different species tested. Differences in experimental procedures and parameters evaluated might also explain the discrepancies. For instance, present study provides the first evidence of the treatment with an AT_1_ receptor antagonist in stress-evoked coat state deterioration. Besides, effect of these drugs in depression-like state evoked by homotypic stressors has never been investigated previously. However, differences in the AT_1_ receptor antagonists employed in the different studies seem not to explain the different findings. For instance, the *ki* values of the several AT_1_ receptor antagonists are equivalent (Alexander et al., 2017), and the dose of losartan used in the present study (30 mg/kg) is similar to those of valsartan (5–40 mg/kg) and irbesartan (40 mg/kg) employed previously (Ping et al., 2014; Ayyub et al., 2016). Besides, lipophilicity and brain penetration of these antagonists do not differ (Michel et al., 2013). Finally, although irbesartan and valsartan exhibited insurmountable antagonism and losartan evoked surmountable antagonist, the active losartan metabolite EXP3174 presented an insurmountable antagonism (de Gasparo et al., 2000; Michel et al., 2013). Accordingly, previous evidence confirmed the efficacy of the dose of losartan used in the present study (see discussion below). However, it is important to mention that treatment in previous studies was longer (28 days) (Ping et al., 2014; Ayyub et al., 2016) in relation to that employed in the present study (10 days), which may contribute to discrepancies.

We also identified that treatment with losartan did not affect the decreased discrimination rates in the NOR test evoked by the chronic stressors. This finding contrasts with previous evidence that systemic treatment with either telmisartan or candesartan inhibited the impairment of short memory evaluated in the NOR test evoked by RRS in rats (Braszko et al., 2013; Wincewicz and Braszko, 2014; Wincewicz et al., 2016). Treatment with telmisartan also inhibited the impairment of spatial memory evoked by RRS in rats (Wincewicz and Braszko, 2015). Although telmisartan is more lipophilic than the other antagonists [i.e., it should be better able to penetrate central nervous system (CNS)], lipophilicity and brain penetration of candesartan and losartan/EXP3174 are similar (Michel et al., 2013). Type of antagonism also seems not to explain the different findings once despite candesartan exhibited insurmountable antagonism, and telmisartan and losartan evoked surmountable antagonist (de Gasparo et al., 2000; Michel et al., 2013). Therefore, an important difference that might explain the discrepancy is treatment time, which was longer (21 days) in previous studies in relation to the present report (10 days) (Braszko et al., 2013; Wincewicz and Braszko, 2014; Wincewicz et al., 2016). Besides, previous studies employed longer protocols of RRS (21 days vs 10 days) with longer restraint sessions (2–2.5 h vs 1 h) (Braszko et al., 2013; Wincewicz and Braszko, 2014; Wincewicz et al., 2016). To the best of our knowledge, present study provides the first evidence of the treatment with an AT_1_ receptor antagonist in CVS-evoked memory impairment.

Previous studies reported dose-dependent binding of losartan to the AT_1_ receptor in brain areas within the blood–brain barrier following peripheral administration of doses ranging from 1 to 100 mg/kg (Song et al., 1991; Zhuo et al., 1994; Wang et al., 2003). These findings are further supported by functional evidence that losartan administrated peripherally (at similar doses to that used in the present study) inhibited the pressor response, water intake, and vasopressin release in the circulation evoked by intracerebroventricular administration of Ang II (Polidori et al., 1996; Culman et al., 1999). Systemic administration of losartan at the same dose used in the present study also evoked antidepressant-like effect in nonstressed animals (Diniz et al., 2018). Furthermore, stress is a condition that might promote increase in blood–brain barrier (BBB) permeability (Skultétyová et al., 1998), thus facilitating the penetration of drugs within the brain. Therefore, the absence of effect of the pharmacological treatment with losartan in depressive-like state and memory impairment evoked by RRS and CVS in the present does not seem to be due to infectivity of the pharmacological treatment. Accordingly, we reported recently that the same treatment with losartan prevented the cardiovascular and autonomic changes evoked by either RRS or CVS (Costa-Ferreira et al., 2016).

Since the behavioral tests employed in the present study (i.e., NOR and sucrose preference) might be influenced by changes in exploratory behavior, the effect of the chronic stressors and the losartan treatment on the locomotor activity in the OF test was also evaluated. Although the findings regarding the effect of CVS in the OF are controversial (Albonetti and Farabollini, 1992; Katz et al., 1981; D’Aquila et al., 2000; Song et al., 2018), our results are in line with previous evidence that this heterotypic stressor increases locomotor activity (Harris, 1997; Grønli et al., 2005). The absence of change following RRS has been proposed to be related to the habituation process (Albonetti and Farabollini, 1992). The CVS-evoked hyperlocomotion is not related to depressive-like state and memory impairment, since a decrease rather than an increase in behavioral responses was identified in sucrose preference and NOR test in animals subjected to the CVS. Interestingly, losartan treatment inhibited the increase in locomotion evoked by CVS, which is in line with previous evidence that irbesartan inhibited the CVS-evoked hypolocomotion in the OF (Ayyub et al., 2016). Besides, it confirms the efficacy of the pharmacological treatment with losartan.

The results reported in the present study also indicated that our treatment with losartan decreased the locomotor activity and impaired the long-term (but not the short-term) memory independently of the stress exposure. Previous studies have already shown that treatment with losartan for either 10 days or 4 or 9 weeks decreased the exploratory activity (Pechlivanova et al., 2011; Tchekalarova et al., 2014; Tchekalarova et al., 2016), thus supporting our findings. Regarding the effects of AT_1_ receptor antagonists in memory, the data are controversial, with some results indicating improvement while others did not identify effects (Braszko, 2005; Braszko et al., 2013; Wincewicz and Braszko, 2014; Wincewicz and Braszko, 2015; Wincewicz et al., 2016; ). Therefore, our data are in line with previous evidence that losartan might impair non-emotional memory.

In summary, the present findings provide evidence that chronic treatment with losartan does not affect the depressive-like state and memory impairment evoked by either homotypic or heterotypic chronic stress regimens in rats. Nevertheless, our results suggest that losartan inhibits hyperlocomotion evoked by heterotypic stressors. Importantly, this study indicates the necessity of further studies evaluating the efficacy of AT_1_ receptor antagonists in treatment of stress-evoked dysfunctions. Indeed, more evidence comparing species (e.g., rats vs mice), stress protocols (e.g., homotypic vs heterotypic), treatment time, and AT_1_ receptor antagonists (e.g., surmountable vs insurmountable antagonists) is necessary to provide more conclusive information regarding the efficacy of AT_1_ receptor antagonists in the treatment of stress-evoked depressive-like state and memory impairment.

## Data Availability Statement

All datasets generated for this study are included in the manuscript and the supplementary files.

## Ethics Statement

This study was carried out in accordance with the recommendations of Brazilian and international guidelines. The protocol was approved by the Ethical Committee for Use of Animals of the School of Pharmaceutical Sciences—UNESP.

## Author Contributions

WC-F and CC contributed to the conception and design of the work. WC-F, GM-S, LG-S, MM, and CC contributed to the acquisition, analysis, and interpretation of data. WC-F and CC drafted the manuscript. GM-S, LG-S and MM critically revised the manuscript, and CC approved the final version to be published.

## Conflict of Interest Statement

The authors declare that the research was conducted in the absence of any commercial or financial relationships that could be construed as a potential conflict of interest.
